# Supplementary material for: Hot spot prediction in protein-protein interactions by an ensemble system
Source: BMC Syst Biol. 2018 Dec 31;12(Suppl 9):132. doi: 10.1186/s12918-018-0665-8 (PMC6311905; doi:10.1186/s12918-018-0665-8)
Supplement: Supplementary file 2 — All relevant MATLAB code. A simple Matlab implement of our predictor. (ZIP 19,742 kb) [file 12918_2018_665_MOESM2_ESM.zip › Additional file 2/Read ASA/ASA website/dbMPIKT/4P4QA.html]

 NetSurfP - Protein Surface Accessibility and Secondary Structure Predictions

  

|  |  |  |
| --- | --- | --- |
|  |  | NetSurfP - Protein Surface Accessibility and Secondary Structure Predictions  Technical University of Denmark |

  


---

```
# For publication of results, please cite:
# A generic method for assignment of reliability scores applied to solvent accessibility predictions.
# Bent Petersen, Thomas Nordahl Petersen, Pernille Andersen, Morten Nielsen and Claus Lundegaard
# BMC Structural Biology 2009, 9:51 doi:10.1186/1472-6807-9-51
#
# Column 1: Class assignment - B for buried or E for Exposed - Threshold: 25% exposure, but not based on RSA
# Column 2: Amino acid
# Column 3: Sequence name
# Column 4: Amino acid number
# Column 5: Relative Surface Accessibility - RSA
# Column 6: Absolute Surface Accessibility
# Column 7: Z-fit score for RSA prediction
# Column 8: Probability for Alpha-Helix
# Column 9: Probability for Beta-strand
# Column 10: Probability for Coil
E T  4P4QA                  1    0.810 112.333  -1.135   0.003   0.003   0.994
E T  4P4QA                  2    0.415  57.533  -0.683   0.052   0.084   0.864
B P  4P4QA                  3    0.288  40.924  -0.864   0.118   0.150   0.732
B L  4P4QA                  4    0.206  37.719  -0.117   0.125   0.227   0.648
B V  4P4QA                  5    0.186  28.573  -1.487   0.135   0.317   0.548
E H  4P4QA                  6    0.390  71.032  -0.317   0.135   0.317   0.548
B V  4P4QA                  7    0.173  26.590   0.011   0.135   0.317   0.548
B A  4P4QA                  8    0.177  19.516  -1.093   0.118   0.150   0.732
E S  4P4QA                  9    0.354  41.430  -0.450   0.191   0.086   0.723
B V  4P4QA                 10    0.242  37.195   0.268   0.191   0.086   0.723
E E  4P4QA                 11    0.412  71.959  -1.187   0.184   0.043   0.773
E K  4P4QA                 12    0.447  92.030  -1.424   0.115   0.016   0.868
E G  4P4QA                 13    0.327  25.766  -1.099   0.058   0.017   0.925
E R  4P4QA                 14    0.621 142.186   0.286   0.058   0.017   0.925
E S  4P4QA                 15    0.289  33.894  -0.540   0.058   0.017   0.925
B Y  4P4QA                 16    0.106  22.588   0.857   0.109   0.005   0.886
E E  4P4QA                 17    0.524  91.578  -0.357   0.246   0.004   0.750
E D  4P4QA                 18    0.465  67.050   0.985   0.406   0.004   0.590
B F  4P4QA                 19    0.037   7.486   0.327   0.970   0.001   0.030
E Q  4P4QA                 20    0.392  69.958   1.426   0.970   0.001   0.030
E K  4P4QA                 21    0.466  95.815   1.761   0.970   0.001   0.030
B V  4P4QA                 22    0.028   4.273   0.936   0.970   0.001   0.030
B Y  4P4QA                 23    0.079  16.818   1.475   0.970   0.001   0.030
E N  4P4QA                 24    0.495  72.409   1.887   0.970   0.001   0.030
E A  4P4QA                 25    0.327  36.057   1.562   0.970   0.001   0.030
B I  4P4QA                 26    0.020   3.700   1.288   0.988   0.000   0.012
B A  4P4QA                 27    0.141  15.571   1.148   0.988   0.000   0.012
E L  4P4QA                 28    0.489  89.627   1.904   0.970   0.001   0.030
B K  4P4QA                 29    0.137  28.181   1.122   0.970   0.001   0.030
B L  4P4QA                 30    0.043   7.855   0.413   0.923   0.002   0.076
E R  4P4QA                 31    0.400  91.669   1.015   0.858   0.002   0.139
E E  4P4QA                 32    0.551  96.347   0.161   0.600   0.003   0.397
E D  4P4QA                 33    0.298  42.913  -0.621   0.246   0.004   0.750
E D  4P4QA                 34    0.415  59.830  -0.781   0.176   0.004   0.820
E E  4P4QA                 35    0.535  93.517   0.129   0.257   0.016   0.727
B Y  4P4QA                 36    0.153  32.611  -0.382   0.339   0.016   0.645
E D  4P4QA                 37    0.392  56.530  -1.303   0.339   0.016   0.645
E N  4P4QA                 38    0.631  92.364  -1.371   0.257   0.016   0.727
B Y  4P4QA                 39    0.277  59.238  -0.621   0.115   0.016   0.868
E I  4P4QA                 40    0.253  46.768  -0.531   0.115   0.016   0.868
B G  4P4QA                 41    0.212  16.669  -1.359   0.113   0.043   0.844
B Y  4P4QA                 42    0.052  11.006   0.162   0.278   0.093   0.628
B G  4P4QA                 43    0.026   2.086   0.315   0.354   0.048   0.598
B P  4P4QA                 44    0.038   5.435  -0.463   0.605   0.105   0.290
B V  4P4QA                 45    0.051   7.900  -0.902   0.649   0.163   0.188
B L  4P4QA                 46    0.020   3.735   1.050   0.662   0.225   0.113
B V  4P4QA                 47    0.018   2.705   1.329   0.594   0.285   0.121
B R  4P4QA                 48    0.045  10.282   0.709   0.594   0.285   0.121
B L  4P4QA                 49    0.018   3.314   1.319   0.594   0.285   0.121
B A  4P4QA                 50    0.025   2.799   0.862   0.478   0.309   0.214
B W  4P4QA                 51    0.027   6.469   0.331   0.352   0.332   0.316
B H  4P4QA                 52    0.029   5.311   0.387   0.321   0.252   0.427
B I  4P4QA                 53    0.031   5.661  -0.512   0.199   0.152   0.649
B S  4P4QA                 54    0.015   1.746   1.148   0.199   0.152   0.649
B G  4P4QA                 55    0.033   2.613  -0.479   0.135   0.317   0.548
B T  4P4QA                 56    0.026   3.592  -0.041   0.135   0.317   0.548
B W  4P4QA                 57    0.156  37.518   0.398   0.064   0.216   0.721
B D  4P4QA                 58    0.209  30.146  -0.696   0.018   0.088   0.893
B K  4P4QA                 59    0.090  18.451  -0.312   0.018   0.088   0.893
E H  4P4QA                 60    0.254  46.275  -0.888   0.052   0.084   0.864
B D  4P4QA                 61    0.151  21.788  -0.960   0.052   0.084   0.864
B N  4P4QA                 62    0.129  18.944  -1.933   0.018   0.088   0.893
B T  4P4QA                 63    0.105  14.522  -0.328   0.021   0.279   0.699
B G  4P4QA                 64    0.056   4.407  -1.025   0.022   0.359   0.619
B G  4P4QA                 65    0.039   3.038  -1.055   0.022   0.359   0.619
B S  4P4QA                 66    0.121  14.205  -1.097   0.020   0.205   0.775
E Y  4P4QA                 67    0.366  78.214  -1.674   0.018   0.047   0.935
E G  4P4QA                 68    0.387  30.488  -1.719   0.018   0.019   0.964
B G  4P4QA                 69    0.191  15.000  -3.014   0.058   0.017   0.925
B T  4P4QA                 70    0.244  33.787  -2.075   0.053   0.043   0.903
B Y  4P4QA                 71    0.296  63.170  -2.058   0.053   0.043   0.903
E R  4P4QA                 72    0.320  73.280  -0.935   0.053   0.043   0.903
B F  4P4QA                 73    0.278  55.775  -1.789   0.053   0.043   0.903
E K  4P4QA                 74    0.380  78.248  -0.535   0.113   0.043   0.844
E K  4P4QA                 75    0.258  53.153  -0.751   0.184   0.043   0.773
B E  4P4QA                 76    0.039   6.813  -2.865   0.184   0.043   0.773
B F  4P4QA                 77    0.261  52.483  -0.650   0.184   0.043   0.773
E N  4P4QA                 78    0.359  52.499  -1.336   0.115   0.016   0.868
B D  4P4QA                 79    0.217  31.327  -0.753   0.115   0.016   0.868
E P  4P4QA                 80    0.283  40.200  -1.010   0.115   0.016   0.868
E S  4P4QA                 81    0.384  44.993  -0.540   0.115   0.016   0.868
B N  4P4QA                 82    0.079  11.595  -3.375   0.115   0.016   0.868
E A  4P4QA                 83    0.315  34.746  -0.118   0.115   0.016   0.868
E G  4P4QA                 84    0.239  18.817  -0.927   0.115   0.016   0.868
B L  4P4QA                 85    0.049   8.972   0.170   0.694   0.003   0.303
E Q  4P4QA                 86    0.468  83.549   0.749   0.858   0.002   0.139
E N  4P4QA                 87    0.364  53.231   1.676   0.858   0.002   0.139
B G  4P4QA                 88    0.015   1.196   0.857   0.970   0.001   0.030
B F  4P4QA                 89    0.110  22.137   0.735   0.970   0.001   0.030
E K  4P4QA                 90    0.463  95.177   1.748   0.970   0.001   0.030
B F  4P4QA                 91    0.051  10.175   0.499   0.970   0.001   0.030
B L  4P4QA                 92    0.024   4.449   0.973   0.970   0.001   0.030
E E  4P4QA                 93    0.330  57.581   1.179   0.970   0.001   0.030
E P  4P4QA                 94    0.434  61.542   1.283   0.923   0.002   0.076
B I  4P4QA                 95    0.030   5.457   0.402   0.923   0.002   0.076
E H  4P4QA                 96    0.266  48.458   0.631   0.858   0.002   0.139
E K  4P4QA                 97    0.612 125.950   0.501   0.858   0.002   0.139
E E  4P4QA                 98    0.363  63.399   0.813   0.782   0.003   0.216
B F  4P4QA                 99    0.158  31.711   0.845   0.321   0.003   0.675
E P  4P4QA                100    0.574  81.436  -0.868   0.053   0.005   0.942
E W  4P4QA                101    0.468 112.530  -0.606   0.018   0.019   0.964
B I  4P4QA                102    0.127  23.569  -0.021   0.053   0.043   0.903
B S  4P4QA                103    0.126  14.791  -0.821   0.115   0.016   0.868
B S  4P4QA                104    0.097  11.345   0.534   0.879   0.010   0.111
B G  4P4QA                105    0.179  14.111  -0.657   0.858   0.002   0.139
B D  4P4QA                106    0.144  20.750   0.256   0.938   0.007   0.055
B L  4P4QA                107    0.018   3.277   1.173   0.938   0.007   0.055
B F  4P4QA                108    0.039   7.888   0.492   0.938   0.007   0.055
B S  4P4QA                109    0.059   6.962  -0.623   0.879   0.010   0.111
B L  4P4QA                110    0.054   9.942   0.031   0.879   0.010   0.111
B G  4P4QA                111    0.022   1.708   0.531   0.879   0.010   0.111
B G  4P4QA                112    0.033   2.629  -0.392   0.802   0.014   0.185
B V  4P4QA                113    0.129  19.750  -0.243   0.879   0.010   0.111
B T  4P4QA                114    0.119  16.436   0.401   0.879   0.010   0.111
B A  4P4QA                115    0.015   1.620   0.916   0.938   0.007   0.055
B V  4P4QA                116    0.045   6.993   0.089   0.938   0.007   0.055
E Q  4P4QA                117    0.304  54.277   0.316   0.879   0.010   0.111
B E  4P4QA                118    0.235  41.142  -0.534   0.831   0.044   0.125
B M  4P4QA                119    0.124  24.792  -1.042   0.339   0.016   0.645
E Q  4P4QA                120    0.313  55.884  -1.886   0.016   0.005   0.979
B G  4P4QA                121    0.185  14.559  -1.257   0.005   0.015   0.979
B P  4P4QA                122    0.284  40.356  -0.551   0.005   0.045   0.951
E K  4P4QA                123    0.346  71.152  -1.239   0.005   0.336   0.660
B I  4P4QA                124    0.093  17.187   0.314   0.004   0.420   0.576
E P  4P4QA                125    0.437  61.954  -0.676   0.022   0.359   0.619
B W  4P4QA                126    0.252  60.654  -0.181   0.020   0.205   0.775
E R  4P4QA                127    0.349  79.829  -1.166   0.018   0.088   0.893
B C  4P4QA                128    0.188  26.451  -0.764   0.018   0.047   0.935
B G  4P4QA                129    0.082   6.414  -2.869   0.018   0.047   0.935
B R  4P4QA                130    0.143  32.839  -0.344   0.018   0.088   0.893
E V  4P4QA                131    0.420  64.477  -0.198   0.052   0.084   0.864
E D  4P4QA                132    0.279  40.175  -0.800   0.053   0.043   0.903
B T  4P4QA                133    0.204  28.267  -1.169   0.113   0.043   0.844
E P  4P4QA                134    0.403  57.228  -0.708   0.184   0.043   0.773
E E  4P4QA                135    0.422  73.741  -0.393   0.184   0.043   0.773
E D  4P4QA                136    0.327  47.077  -2.552   0.184   0.043   0.773
E T  4P4QA                137    0.398  55.189  -0.746   0.184   0.043   0.773
B T  4P4QA                138    0.279  38.725  -1.085   0.184   0.043   0.773
E P  4P4QA                139    0.425  60.336  -0.358   0.181   0.016   0.803
E D  4P4QA                140    0.607  87.454  -1.590   0.181   0.016   0.803
E N  4P4QA                141    0.541  79.173  -1.310   0.115   0.016   0.868
E G  4P4QA                142    0.614  48.290  -2.062   0.018   0.019   0.964
E R  4P4QA                143    0.382  87.386  -0.713   0.018   0.019   0.964
B L  4P4QA                144    0.116  21.221   0.066   0.018   0.019   0.964
B P  4P4QA                145    0.088  12.487  -1.338   0.003   0.003   0.994
E D  4P4QA                146    0.333  47.913  -0.165   0.005   0.015   0.979
B A  4P4QA                147    0.193  21.236  -1.072   0.005   0.015   0.979
E D  4P4QA                148    0.508  73.246  -1.184   0.018   0.019   0.964
B K  4P4QA                149    0.257  52.906  -1.113   0.018   0.019   0.964
E D  4P4QA                150    0.415  59.729   0.077   0.053   0.005   0.942
B A  4P4QA                151    0.067   7.394  -0.351   0.923   0.002   0.076
E G  4P4QA                152    0.576  45.363   1.282   0.923   0.002   0.076
E Y  4P4QA                153    0.389  83.065   1.507   0.923   0.002   0.076
B V  4P4QA                154    0.028   4.273   0.769   0.970   0.001   0.030
B R  4P4QA                155    0.198  45.296   1.367   0.970   0.001   0.030
E T  4P4QA                156    0.486  67.478   1.001   0.970   0.001   0.030
B F  4P4QA                157    0.170  34.079   1.486   0.970   0.001   0.030
B F  4P4QA                158    0.037   7.386  -0.954   0.970   0.001   0.030
E Q  4P4QA                159    0.395  70.529   0.519   0.923   0.002   0.076
E R  4P4QA                160    0.641 146.881  -0.351   0.782   0.003   0.216
E L  4P4QA                161    0.324  59.269   1.010   0.246   0.004   0.750
E N  4P4QA                162    0.607  88.821  -0.251   0.016   0.005   0.979
B M  4P4QA                163    0.068  13.627  -0.121   0.003   0.003   0.994
E N  4P4QA                164    0.407  59.658   0.992   0.016   0.005   0.979
E D  4P4QA                165    0.300  43.158  -0.089   0.354   0.048   0.598
E R  4P4QA                166    0.399  91.279   1.183   0.354   0.048   0.598
B E  4P4QA                167    0.109  19.095   0.313   0.386   0.097   0.517
B V  4P4QA                168    0.029   4.473   1.057   0.352   0.332   0.316
B V  4P4QA                169    0.029   4.473   0.299   0.273   0.587   0.140
B A  4P4QA                170    0.042   4.672   0.135   0.265   0.667   0.069
B L  4P4QA                171    0.022   4.065   0.797   0.273   0.587   0.140
B M  4P4QA                172    0.029   5.823   0.553   0.268   0.505   0.227
B G  4P4QA                173    0.098   7.705  -0.753   0.135   0.317   0.548
B A  4P4QA                174    0.066   7.262  -1.245   0.118   0.150   0.732
B H  4P4QA                175    0.086  15.607  -0.192   0.125   0.227   0.648
B A  4P4QA                176    0.085   9.400  -0.655   0.064   0.216   0.721
B L  4P4QA                177    0.082  14.923  -0.923   0.066   0.296   0.638
B G  4P4QA                178    0.079   6.194  -2.530   0.064   0.216   0.721
E K  4P4QA                179    0.320  65.742  -0.218   0.064   0.216   0.721
B T  4P4QA                180    0.040   5.603  -1.573   0.064   0.216   0.721
B H  4P4QA                181    0.140  25.375   0.001   0.066   0.296   0.638
B L  4P4QA                182    0.182  33.269  -0.928   0.021   0.279   0.699
E K  4P4QA                183    0.314  64.508  -0.929   0.020   0.205   0.775
B N  4P4QA                184    0.235  34.419  -1.723   0.018   0.047   0.935
B S  4P4QA                185    0.215  25.233  -2.362   0.018   0.019   0.964
E G  4P4QA                186    0.312  24.578  -2.137   0.018   0.047   0.935
B Y  4P4QA                187    0.196  41.800  -0.729   0.018   0.088   0.893
E E  4P4QA                188    0.476  83.140  -1.312   0.018   0.088   0.893
B G  4P4QA                189    0.225  17.684  -2.558   0.018   0.047   0.935
E P  4P4QA                190    0.382  54.177  -1.689   0.018   0.088   0.893
E F  4P4QA                191    0.313  62.759  -2.291   0.018   0.047   0.935
E G  4P4QA                192    0.445  35.045  -1.171   0.018   0.019   0.964
E A  4P4QA                193    0.565  62.285  -2.191   0.018   0.019   0.964
E A  4P4QA                194    0.548  60.445  -1.370   0.058   0.017   0.925
E N  4P4QA                195    0.316  46.233  -1.217   0.058   0.017   0.925
B N  4P4QA                196    0.266  38.972  -0.971   0.018   0.019   0.964
E V  4P4QA                197    0.423  65.077  -0.686   0.058   0.017   0.925
B F  4P4QA                198    0.078  15.554  -1.786   0.058   0.017   0.925
B T  4P4QA                199    0.148  20.472  -1.022   0.018   0.019   0.964
B N  4P4QA                200    0.264  38.664  -1.496   0.502   0.002   0.495
E E  4P4QA                201    0.489  85.446  -1.066   0.694   0.003   0.303
B F  4P4QA                202    0.086  17.220  -0.186   0.782   0.003   0.216
B Y  4P4QA                203    0.028   5.941  -0.429   0.923   0.002   0.076
E L  4P4QA                204    0.302  55.369   0.391   0.923   0.002   0.076
E N  4P4QA                205    0.315  46.072  -0.316   0.858   0.002   0.139
B L  4P4QA                206    0.033   6.042  -0.507   0.858   0.002   0.139
B L  4P4QA                207    0.093  16.973   0.037   0.782   0.003   0.216
E N  4P4QA                208    0.591  86.464  -0.187   0.502   0.002   0.495
E E  4P4QA                209    0.326  56.970  -0.610   0.109   0.005   0.886
E D  4P4QA                210    0.315  45.420  -1.601   0.058   0.017   0.925
B W  4P4QA                211    0.119  28.644  -1.120   0.113   0.043   0.844
E K  4P4QA                212    0.575 118.195  -1.118   0.113   0.087   0.800
E L  4P4QA                213    0.357  65.422  -0.647   0.052   0.084   0.864
E E  4P4QA                214    0.394  68.762  -1.689   0.053   0.043   0.903
E K  4P4QA                215    0.475  97.646  -1.703   0.018   0.047   0.935
E N  4P4QA                216    0.310  45.384  -1.955   0.018   0.088   0.893
E D  4P4QA                217    0.419  60.349  -1.883   0.018   0.047   0.935
E A  4P4QA                218    0.347  38.195  -2.882   0.018   0.019   0.964
E N  4P4QA                219    0.470  68.793  -1.144   0.018   0.047   0.935
E N  4P4QA                220    0.291  42.661  -1.556   0.064   0.216   0.721
E E  4P4QA                221    0.300  52.445  -0.112   0.021   0.451   0.528
B Q  4P4QA                222    0.175  31.201  -1.351   0.021   0.451   0.528
B W  4P4QA                223    0.220  52.838   0.096   0.022   0.552   0.426
E D  4P4QA                224    0.289  41.587  -0.083   0.021   0.451   0.528
B S  4P4QA                225    0.229  26.815  -0.605   0.066   0.296   0.638
E K  4P4QA                226    0.381  78.372  -0.308   0.056   0.142   0.802
E S  4P4QA                227    0.596  69.828  -0.374   0.052   0.084   0.864
E G  4P4QA                228    0.506  39.814  -1.143   0.018   0.047   0.935
E Y  4P4QA                229    0.281  60.135   0.008   0.019   0.141   0.840
B M  4P4QA                230    0.064  12.786  -1.881   0.022   0.359   0.619
B M  4P4QA                231    0.111  22.231  -0.113   0.022   0.552   0.426
B L  4P4QA                232    0.052   9.540   0.663   0.074   0.484   0.442
B P  4P4QA                233    0.235  33.361  -0.058   0.216   0.235   0.548
B T  4P4QA                234    0.039   5.465  -0.497   0.502   0.102   0.396
B D  4P4QA                235    0.053   7.637  -0.606   0.802   0.014   0.185
E Y  4P4QA                236    0.243  51.886   0.321   0.879   0.010   0.111
B S  4P4QA                237    0.171  20.065   0.319   0.879   0.010   0.111
B L  4P4QA                238    0.038   7.049   0.350   0.802   0.014   0.185
B I  4P4QA                239    0.261  48.303  -0.217   0.622   0.015   0.363
E Q  4P4QA                240    0.511  91.175   0.076   0.181   0.016   0.803
E D  4P4QA                241    0.251  36.198   0.293   0.018   0.019   0.964
E P  4P4QA                242    0.672  95.414  -0.852   0.406   0.004   0.590
E K  4P4QA                243    0.489 100.608   0.571   0.694   0.003   0.303
B Y  4P4QA                244    0.042   9.040  -0.266   0.858   0.002   0.139
B L  4P4QA                245    0.195  35.686   0.989   0.970   0.001   0.030
E S  4P4QA                246    0.505  59.186   1.172   0.970   0.001   0.030
B I  4P4QA                247    0.106  19.591   1.535   0.970   0.001   0.030
B V  4P4QA                248    0.015   2.305   1.429   0.988   0.000   0.012
E K  4P4QA                249    0.388  79.750   1.783   0.970   0.001   0.030
E E  4P4QA                250    0.400  69.897   1.451   0.975   0.003   0.022
B Y  4P4QA                251    0.071  15.258   0.623   0.938   0.007   0.055
B A  4P4QA                252    0.153  16.806  -0.908   0.694   0.003   0.303
E N  4P4QA                253    0.639  93.564   0.641   0.246   0.004   0.750
E D  4P4QA                254    0.339  48.807   0.156   0.016   0.005   0.979
E Q  4P4QA                255    0.330  58.938   0.225   0.782   0.003   0.216
E D  4P4QA                256    0.624  89.976   1.005   0.923   0.002   0.076
E K  4P4QA                257    0.344  70.802   2.198   0.970   0.001   0.030
B F  4P4QA                258    0.034   6.804   0.570   0.970   0.001   0.030
B F  4P4QA                259    0.166  33.216   0.704   0.970   0.001   0.030
E K  4P4QA                260    0.519 106.820   1.351   0.970   0.001   0.030
B D  4P4QA                261    0.225  32.451   1.669   0.970   0.001   0.030
B F  4P4QA                262    0.030   6.041   0.983   0.988   0.000   0.012
B S  4P4QA                263    0.070   8.251   0.272   0.988   0.000   0.012
E K  4P4QA                264    0.384  79.009   1.906   0.988   0.000   0.012
B A  4P4QA                265    0.031   3.427   0.293   0.988   0.000   0.012
B F  4P4QA                266    0.053  10.617   0.686   0.988   0.000   0.012
B E  4P4QA                267    0.167  29.227   0.188   0.970   0.001   0.030
B K  4P4QA                268    0.200  41.058   1.030   0.938   0.007   0.055
B L  4P4QA                269    0.041   7.544   0.233   0.879   0.010   0.111
B L  4P4QA                270    0.105  19.299  -1.379   0.717   0.014   0.269
E E  4P4QA                271    0.453  79.157  -0.364   0.268   0.043   0.689
B N  4P4QA                272    0.091  13.249   0.407   0.052   0.084   0.864
E G  4P4QA                273    0.441  34.675  -0.908   0.018   0.047   0.935
B I  4P4QA                274    0.103  19.036  -0.564   0.018   0.047   0.935
E T  4P4QA                275    0.323  44.856  -0.538   0.018   0.088   0.893
B F  4P4QA                276    0.314  63.100  -0.817   0.018   0.047   0.935
E P  4P4QA                277    0.332  47.111  -1.981   0.018   0.019   0.964
E K  4P4QA                278    0.445  91.557  -1.913   0.018   0.019   0.964
E D  4P4QA                279    0.495  71.286  -1.152   0.018   0.019   0.964
B A  4P4QA                280    0.276  30.415  -1.217   0.018   0.019   0.964
E P  4P4QA                281    0.470  66.693  -1.069   0.018   0.019   0.964
E S  4P4QA                282    0.465  54.510  -0.953   0.018   0.047   0.935
E P  4P4QA                283    0.319  45.309  -1.429   0.018   0.088   0.893
B F  4P4QA                284    0.224  44.997  -0.627   0.022   0.359   0.619
E I  4P4QA                285    0.239  44.271   0.186   0.021   0.451   0.528
B F  4P4QA                286    0.212  42.488   0.305   0.021   0.451   0.528
E K  4P4QA                287    0.451  92.709   0.437   0.021   0.279   0.699
E T  4P4QA                288    0.359  49.821  -0.682   0.018   0.088   0.893
B L  4P4QA                289    0.193  35.357   0.437   0.018   0.019   0.964
E E  4P4QA                290    0.674 117.783   0.350   0.018   0.047   0.935
E E  4P4QA                291    0.533  93.028  -0.804   0.053   0.043   0.903
E Q  4P4QA                292    0.574 102.445  -0.890   0.058   0.017   0.925
E G  4P4QA                293    0.654  51.462  -1.896   0.018   0.019   0.964
E L  4P4QA                294    0.537  98.398  -1.752   0.003   0.003   0.994

Explain the output.  Go back.
```

---
